# Modern treatment for achalasia: endoscopic and surgical therapies

**DOI:** 10.1093/bjs/znag046

**Published:** 2026-04-27

**Authors:** Andrew Conner, Melissa V Wills, Salvador Navarrete, Jerry T Dang, Robert Bechara, Biniam Kidane, Siva Raja, Matthew Kroh, Dennis Hong, Yung Lee

**Affiliations:** Department of General Surgery, Digestive Disease Institute, Cleveland Clinic, Cleveland, Ohio, USA; Department of Thoracic and Cardiovascular Surgery, Heart, Vascular, and Thoracic Institute, Cleveland Clinic, Cleveland, Ohio, USA; Department of General Surgery, Digestive Disease Institute, Cleveland Clinic, Cleveland, Ohio, USA; Department of General Surgery, Digestive Disease Institute, Cleveland Clinic, Cleveland, Ohio, USA; Department of General Surgery, Digestive Disease Institute, Cleveland Clinic, Cleveland, Ohio, USA; Division of Gastroenterology, Queen’s University, Kingston, Ontario, Canada; Section of Thoracic Surgery, University of Manitoba, Winnipeg, Manitoba, Canada; Department of Thoracic and Cardiovascular Surgery, Heart, Vascular, and Thoracic Institute, Cleveland Clinic, Cleveland, Ohio, USA; Department of General Surgery, Digestive Disease Institute, Cleveland Clinic, Cleveland, Ohio, USA; Division of General Surgery, Department of Surgery, McMaster University, Hamilton, Ontario, Canada; Department of General Surgery, Digestive Disease Institute, Cleveland Clinic, Cleveland, Ohio, USA

## Abstract

Achalasia is a rare, progressive oesophageal motility disorder defined by impaired lower oesophageal sphincter relaxation and absent peristalsis, leading to dysphagia, regurgitation, chest pain, weight loss, and increased long-term risks of aspiration and malignancy. Management has evolved from open surgical myotomy to minimally invasive laparoscopic and robotic techniques and, more recently, peroral endoscopic myotomy (POEM). This review summarizes contemporary diagnostic strategies, including high-resolution manometry, timed barium oesophagram, endoscopy, and emerging applications of impedance planimetry, and critically appraises current endoscopic and surgical therapies. The review compares outcomes of pneumatic dilation, botulinum toxin injection, minimally invasive Heller myotomy with fundoplication, POEM, POEM with fundoplication, and newer approaches for advanced disease such as peroral oesophageal plication and oesophagectomy, integrating data from randomized trials and long-term cohort studies. Key issues, including post-treatment gastro-oesophageal reflux, cancer surveillance, and management of recurrent or refractory symptoms, are addressed. Treatment selection is emphasized as individualized, incorporating manometric subtype, oesophageal morphology, patient co-morbidity, institutional expertise, procedural durability, complication profiles, and evolving guideline recommendations across international expert consensus groups. Contemporary multimodal therapy enables durable symptom control and meaningful quality-of-life improvement for most patients, while ongoing innovation and longer-term follow-up will continue to refine treatment algorithms and standards of care.

## Introduction

Achalasia is a rare, progressive oesophageal motility disorder with a global incidence of approximately 0.78 per 100 000 person-years and a prevalence of 100 000 persons, though adoption of high-resolution manometry has yielded higher estimates in some regions (2.3–2.9 per 100 000 person-years)^1–3^. Incidence varies geographically—2.92 per 100 000 in Central Chicago, 2.3–2.8 in South Australia, 1.99 in England, and 0.81–1.37 in Japan—likely reflecting differences in diagnostic access rather than true epidemiological variation^1–5^. The disease occurs equally in men and women and across racial groups^6–8^. The incidence rises with age from 10.5 per 100 000 in those aged <65 years to 26.0 per 100 000 in those aged ≥65 years^9^.

Achalasia results from selective loss of inhibitory neurones within the myenteric plexus of the distal oesophagus and of the lower oesophageal sphincter (LOS), disrupting the balance between excitatory (acetylcholine) and inhibitory (nitric oxide, vasoactive intestinal peptide) neurotransmission^6–8^. Current evidence strongly supports that achalasia is an autoimmune disease involving both cell-mediated and antibody-mediated immune responses against myenteric neurones^8,10^. The first achalasia genomic-wide association study published in 2025 found that genetic risk is largely mediated through immune pathways—most predominantly through HLA class II variants^11^. Familial clustering in twins and first-degree relatives further supports a heritable component^8,12^. The prevailing hypothesis is that viral infection triggers autoimmune destruction in genetically susceptible individuals^13,14^. Proposed viral triggers include herpes simplex virus 1, varicella zoster virus, human papillomavirus, and measles virus. A large case–control study found that the presence of any viral infection was associated with a 1.58-fold increased odds of achalasia^15^.

These mechanism culminate in failure of the LOS to relax appropriately during swallowing and loss of normal oesophageal peristalsis^6,7,16^. Patients typically experience progressive dysphagia to solids and liquids, regurgitation of undigested food, chest pain, and weight loss—symptoms quantified by the validated Eckardt score (total score: 0–12, with higher scores indicating greater severity) (*Table S1*)^17^. An Eckardt score of ≤3 is often defined as clinical remission and treatment success in the literature. Without intervention, the disease progresses and does not spontaneously remit^8,16^, and the oesophagus undergoes gradual dilation due to functional obstruction, with approximately 5% of patients developing end-stage sigmoid morphology^18^. Disease progression and treatment response are influenced by disease subtype, which is based on oesophageal pressurization patterns: failed peristalsis without pressurization (type I); absent peristalsis with pan-oesophageal pressurization (type II); and spastic, luminal-obliterating contractions (type III). Type II, the most common, carries the best prognosis with treatment and likely represents an earlier disease stage, while type I is associated with greater oesophageal dilation suggesting more advanced disease. Whether subtypes represent a true progression continuum remains incompletely established. Long-term complications of achalasia include aspiration pneumonia, malnutrition, and impaired quality of life^6,7,16^.

Treatment aims to improve oesophageal emptying, palliate symptoms, and delay disease progression. Over the past century, management has evolved from open Heller myotomy (first described in 1913 via thoracotomy or laparotomy) to minimally invasive laparoscopic and robotic approaches, and, subsequently, to peroral endoscopic myotomy (POEM)^6,7,16^. This narrative review comprehensively evaluates contemporary endoscopic and surgical treatment modalities for achalasia, compares outcomes, provides evidence-based guidance for treatment selection based on patient and disease factors, and identifies areas for future investigation.

## Diagnosis and evaluation

### Clinical history

Achalasia typically presents insidiously, with symptoms often progressing over months to years before a diagnosis is established, with a mean diagnostic delay of 4–5 years^8,19^. Dysphagia to both solids and liquids is the cardinal symptom, often accompanied by regurgitation, chest pain, and weight loss. This gradual onset distinguishes achalasia from pseudoachalasia, which mimics the manometric and clinical features but results from mechanical obstruction, most commonly malignancy at or near the oesophagogastric junction^8,20^. Pseudoachalasia should be suspected when symptoms are of short duration (<12 months), when weight loss is pronounced (≥10 kg), or when the patient is older at symptom onset (≥55 years), as these features are atypical of achalasia and warrant cross-sectional imaging and endoscopic evaluation to exclude an underlying malignancy^8,21^.

### High-resolution manometry

High-resolution manometry is the ‘gold standard’ for diagnosis with the Chicago Classification defining achalasia as impaired LOS relaxation (integrated relaxation pressure >15 mmHg) and absent normal peristalsis^22^. High-resolution manometry also distinguishes clinically relevant subtypes that guide treatment selection and prognostication.

### Timed barium oesophagram (TBO)

TBO provides complementary functional and morphological assessment of the oesophagus. Patients ingest 250 ml of barium, with chest X-rays obtained at 1 and 5 min to measure oesophageal emptying by barium column height and width measurements^23^. TBO is the preferred first-line test for evaluating persistent or recurrent symptoms after treatment^6^.

### Upper endoscopy

Upper endoscopy is essential to rule out pseudoachalasia caused by external compression from malignancy, stricture, or hiatal hernia. Endoscopic findings of achalasia typically include oesophageal dilation with retained food and saliva, and an oesophagogastric junction with increased resistance to passage of an endoscope. Cross-sectional imaging with CT or endoscopic ultrasonography can be considered, particularly in those with alarm features (for example significant weight loss, symptom duration <12 months, or age ≥55 years at onset) to further exclude malignancy and extrinsic compression as causes of pseudoachalasia.

### Chicago Classification of achalasia subtypes

The Chicago Classification categorizes achalasia into three distinct subtypes based on manometric patterns, each with unique treatment implications. Type I, representing 20–40% of patients, is characterized by 100% failed peristalsis without oesophageal pressurization and minimal contractile activity in the oesophageal body. This subtype demonstrates intermediate treatment outcomes, with ongoing debate regarding the role of fundoplication given the complete absence of oesophageal motor activity in this subtype^24^. Type II achalasia, the most common subtype (50–70% of patients), is defined by pan-oesophageal pressurization >30 mmHg in at least 20% of swallows. This subtype demonstrates the most favourable outcomes across all treatment modalities, with success rates exceeding 90% for surgical myotomy^25^. Type III, the least common variant (5% of patients), is characterized by premature or spastic contractions with obstructing spasms in the distal oesophagus in at least 20% of swallows^6^. The relatively less favourable response to Heller myotomy in type III achalasia—primarily in terms of dysphagia resolution, which drives the composite Eckardt score—has led to POEM emerging as the preferred first-line treatment for this subtype, owing to the ability to perform a longer myotomy extending to the proximal extent of spastic contractions^6,25–27^. Chest pain, however, improves in approximately 70% of patients and does not appear to be significantly influenced by myotomy length^28^.

### Oesophageal morphology

**Fig. 1:**
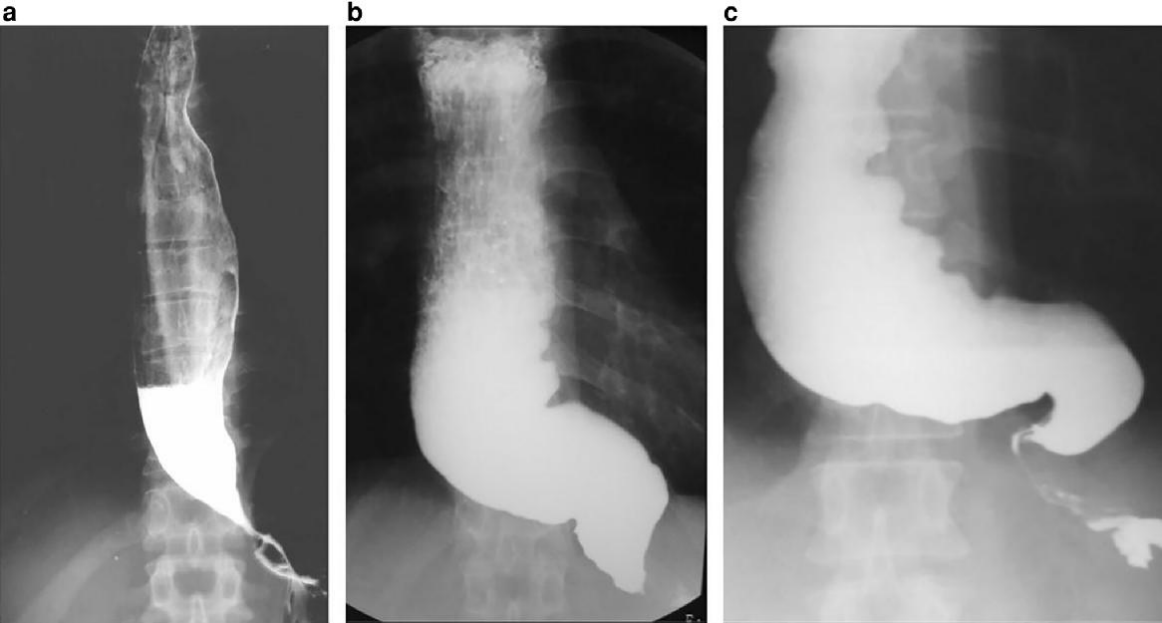
Oesophageal morphology in achalasia Barium oesophagrams demonstrating the morphological spectrum classified by the Japan Esophageal Society^29^. **a** Straight oesophagus with dilation and distal ‘bird-beak’ narrowing. **b** Sigmoid oesophagus with angulation of the distal oesophagus. **c** Advanced sigmoid (sink-trap) oesophagus with massive dilation and severe tortuosity.

Oesophageal morphology provides prognostic information beyond manometric classification. The Japan Esophageal Society classifies morphology by combining dilation with tortuosity (graded as straight, sigmoid, or advanced sigmoid) based on the angle of oesophageal flexion (*Fig. 1*)^29^. However, this system has been adopted infrequently in North America, potentially attributable to the system’s complexity and the continuous spectrum of oesophageal morphology. Although oesophageal dilation can be objectively quantified by oesophageal width measurements, oesophageal tortuosity assessment remains subjective, resulting in substantial inter-observer variability even among experienced clinicians at high-volume centres. The oesophageal length-to-height ratio (LHR), however, objectively quantifies tortuosity, with higher ratios indicating more advanced disease^30^, and is highly predictive of long-term post-myotomy symptom relief and oesophageal emptying^31^. The Chicago Classification provides a framework for treatment selection based on manometric patterns, but does not account for morphological features that also may influence treatment outcomes. How morphology should inform the choice between treatment modalities remains incompletely elucidated and represents an area for future investigation.

## Treatment of achalasia

All treatment modalities for achalasia are directed toward reducing LOS obstruction. The primary aim should be symptom relief, with improvement in objectively measured oesophageal emptying being an important additional treatment goal^32^. Multiple therapeutic approaches exist to achieve these aims, including botulinum toxin injection, pneumatic dilation, and surgical and endoscopic myotomy.

### Botulinum toxin injection

Botulinum toxin type A (onabotulinumtoxinA) inhibits acetylcholine release at nerve terminals, and, when injected into the LOS, reduces sphincter pressure by blocking excitatory neurotransmission^33^. The procedure involves administering 100 units of toxin, divided into 0.5–1 ml aliquots, into four quadrants of the LOS just above the squamocolumnar junction using a sclerotherapy needle during upper endoscopy. This approach ensures even distribution and maximal therapeutic effect. The American College of Gastroenterology recommends a total dose of 100 units, as higher doses have not demonstrated superior efficacy. The procedure is generally very safe with rare serious adverse events^6–8^.

The therapeutic effect is temporary, lasting 3–6 months on average, making botulinum toxin injection suitable only for specific clinical scenarios^34,35^. The primary indication for botulinum toxin injection is for patients who are unfit for more invasive treatments, or in whom a more definite treatment needs to be deferred^32^. However, botulinum toxin injection also serves a diagnostic role in cases of uncertainty and may predict response to more definitive therapy such as myotomy, as a positive response is thought to suggest favourable outcomes with myotomy, though this remains based on clinical experience rather than robust evidence. Repeated injections may complicate subsequent surgery due to submucosal fibrosis, though evidence is conflicting^6^.

### Pneumatic dilation

Pneumatic dilation forcibly disrupts LOS muscle fibres using a graded, stepwise approach with progressively larger balloons (30, 35, and 40 mm) inflated under fluoroscopic guidance. Under sedation, a balloon dilator is positioned across the LOS and inflated to 7–15 psi for 15–60 s until the fluoroscopic waist sign is obliterated. Initial dilation is performed with a 30-mm balloon, with symptom and objective reassessment shortly thereafter. If symptoms persist or LOS pressure remains elevated, subsequent dilations with larger balloons are performed. The procedure typically utilizes this stepwise, graded approach with progressively larger balloons to minimize perforation risk. Initial success rates range from 74% to 86%, though 25% of patients require repeat dilation within 5 years^6,7,36^.

In the current treatment algorithm, pneumatic dilation primarily serves patients who are poor surgical candidates due to significant co-morbidities, those unable to undergo general anaesthesia, those seeking temporizing therapy before more definitive treatment, or those with recurrent symptoms after prior myotomy. Although the European Achalasia Trial demonstrated comparable short- to long-term outcomes between pneumatic dilation and laparoscopic Heller myotomy, this equivalence must be interpreted in light of 25% of pneumatic dilation patients requiring redilation by 5 years, with recurrence rates continuing to rise thereafter^36,37^. The Society of American Gastrointestinal and Endoscopic Surgeons (SAGES) guidelines therefore position pneumatic dilation as inferior to POEM for long-term symptom control, recommending either POEM or laparoscopic Heller myotomy as first-line treatment for type I and II achalasia^38^. For patients ultimately undergoing myotomy, prior pneumatic dilation may complicate laparoscopic Heller myotomy, though evidence is conflicting^39,40^, and does not appear to significantly affect POEM outcomes^41^.

### Minimally invasive Heller myotomy

Heller myotomy with partial fundoplication is the ‘gold standard’ surgical treatment of achalasia. This procedure is predominately performed using minimally invasive approaches. The operation involves dividing the circular muscle fibres of the LOS without disrupting the mucosa. The standard technique includes a myotomy extending at least 5 cm proximal to the gastro-oesophageal junction with extension at least 2 cm onto the gastric cardia^42^. Heller myotomy achieves clinical success in about 90% of patients at 2 years and 78–90% at 5 years, with success defined as an Eckardt score of ≤3 or marked dysphagia improvement^6,7,43–45^. Complete emptying by 5 min on TBO is achieved in 55–70% of patients immediately after Heller, but declines to 24–40% at 5 years^45^. Reinterventions (that is POEM, redo Heller myotomy, pneumatic dilation, oesophagectomy) occur in 5–15% at 2 years, and 18–27% at 5 years, most commonly for recurrent dysphagia, with highest risk within the first postoperative year^7,45^.

Partial fundoplication is routinely added to mitigate postoperative reflux, with two main options available—the anterior Dor or the posterior Toupet fundoplication. A three-stitch modification of the Dor has been described, providing effective reflux control while allowing easier takedown if revision is required^46^. A multicentre randomized trial found a non-significant difference in abnormal pH test results between the Dor and Toupet fundoplication (41% *versus* 21%; *P* = 0.152), with similar improvements in dysphagia and regurgitation^47^. Meta-analysis showed no significant difference in acid exposure between the two approaches, though dysphagia and reinterventions were lower with posterior fundoplication^48^.

As robotic-assisted surgery becomes increasingly utilized, direct comparisons between robotic and laparoscopic Heller myotomy have provided insight into minimally invasive approaches. A recent study analysing 170 robotic and 277 laparoscopic Heller myotomies demonstrated low complication rates, including mucosal perforation rates, for both techniques, with no perforations in the robotic group and a mucosal perforation rate of 1.8% in the laparoscopic group; in addition, both groups had lengths of stay in hospital that were equivalent^49^. Both approaches yield excellent outcomes with durable symptom relief and oesophageal emptying in experienced hands. In propensity-matched analysis of patients with normal oesophageal morphology, the robotic approach had fewer reinterventions (redo myotomy, POEM, pneumatic dilation, oesophagectomy) at 3 years (1.2% *versus* 11%; *P* = 0.04).

### POEM

POEM, introduced by Inoue *et al*.^50^ in 2010 and popularized in Japan, has gained widespread acceptance as a first-line endoscopic treatment option for achalasia. The standard technique involves submucosal injection solution at the mid-oesophagus (approximately 5–6 cm proximal to the gastro-oesophageal junction), followed by a 2 cm longitudinal mucosal incision to access the submucosal space. A submucosal tunnel is created, and the myotomy is performed beginning 3–4 cm distal to the mucosotomy. The myotomy is extended 2–4 cm onto the gastric wall, with contemporary practice favouring adequate gastric extension to minimize risk of incomplete myotomy—a primary cause of treatment failure. The mucosotomy is then closed with endoscopic clips. Compared with surgical myotomy, POEM provides the theoretical advantage of avoiding extensive mediastinal mobilization needed for a longer myotomy and enables the ability to perform a posterior myotomy in patients with prior anterior oesophageal surgery, thereby avoiding scarred surgical planes. Multiple studies demonstrate excellent efficacy with clinical success (Eckardt score of ≤3) achieved in 83–92% of patients at 2 years^6,42,51–56^, and sustained symptom relief in 79–92% at ≥5 years^55–59^. Long-term outcomes show complete oesophageal emptying by 5 min on TBO in 24% at 5 years^60^, and low frequency of reinterventions (4–7%)^56,57^. POEM demonstrates superior outcomes for type III achalasia compared with Heller myotomy^6,25–27^, and is an effective salvage therapy after failed surgical myotomy^60^.

### Post-POEM reflux management and cancer surveillance

The major limitation of POEM is post-procedure gastro-oesophageal reflux disease (GERD) due to the absence of a concurrent anti-reflux procedure. In an RCT comparing POEM with laparoscopic Heller myotomy with Dor fundoplication, Werner *et al*.^42^ demonstrated a significantly higher frequency of reflux oesophagitis in the POEM group at 3 months (57% *versus* 20%; OR 5.74 (95% c.i. 2.99 to 11.00)) and 2 years (44% *versus* 29%; OR 2.00 (95% c.i. 1.03 to 3.85)). Although objective pH testing initially showed higher abnormal reflux in the POEM group at 3 months (44% *versus* 33%), this difference equalized by 2 years (30% in both groups). However, 5-year follow-up data showed sustained differences between groups, with 41% of POEM patients demonstrating reflux oesophagitis and 62% showing abnormal pH testing, compared with 31% for both metrics in the Heller myotomy group^61^. These longitudinal findings reveal a modest temporal decline in oesophagitis after POEM, hypothesized to result from ongoing healing and remodelling of the LOS. Interestingly, a propensity-matched comparison between patients undergoing index POEM and salvage POEM after previous Heller myotomy found similar frequencies of reflux oesophagitis and abnormal acid exposure in the two groups^60^. This finding is notable given that most salvage POEM patients had undergone prior fundoplication. These results suggest that prior fundoplication does not protect against post-POEM reflux, and that post-POEM reflux may be intrinsic to the procedure rather than attributable to the absence of fundoplication^60^. Interpretation of all of the aforementioned findings is complicated by the absence of standardized reflux definitions specifically validated for achalasia patients, as all current diagnostic thresholds are derived from non-achalasia populations with normal oesophageal physiology.

**Fig. 2:**
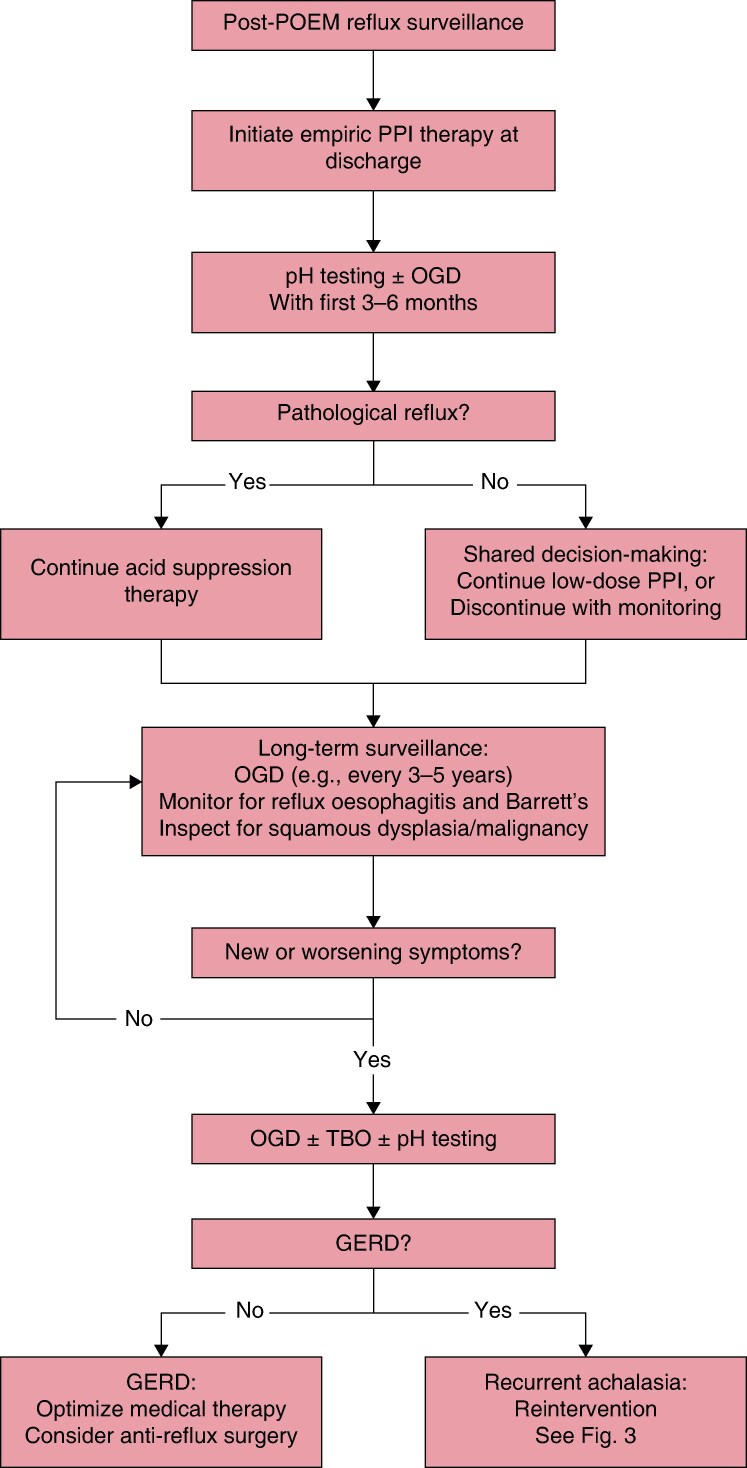
Post-POEM reflux surveillance algorithm Algorithm for empiric acid suppression, objective reflux assessment, and long-term endoscopic surveillance after POEM. Optimal surveillance intervals have not been established; frequency should be individualized based on reflux severity, risk of Barrett’s oesophagus, and institutional protocols. POEM, peroral endoscopic myotomy; PPI, proton pump inhibitor; OGD, oesophagogastroduodenoscopy; TBO, timed barium oesophagram; GERD, gastro-oesophageal reflux disease.

No consensus exists regarding the optimal regimen for empiric acid suppression after POEM, with variation in practice patterns across institutions. However, expert centres generally employ a three-phase approach: immediate post-procedure management, with patients initiated on proton pump inhibitor (PPI) therapy at hospital discharge, typically as twice-daily dosing for at least 2–3 months; surveillance, with objective assessment via pH testing and/or endoscopy performed within the first 3–6 months to guide ongoing management; and long-term therapy, tailored based on objective findings and patient preference. Patients with abnormal pH testing or evidence of reflux oesophagitis are continued on acid suppression therapy. For patients with normal objective testing, a shared decision-making approach can be employed, weighing the risks of long-term PPI use against potential reflux-related complications, with options including continuation of low-dose acid suppression or close symptomatic monitoring off therapy. *Figure 2* outlines a post-POEM reflux surveillance algorithm, including empiric acid suppression, objective assessment, and long-term monitoring Approximately half of patients require PPI therapy after POEM at 5 years^61^. Failure to initiate appropriate acid suppression therapy post-POEM can potentially lead to misdiagnosis of recurrent achalasia, as severe reflux symptoms may mimic disease recurrence. The majority of patients achieve adequate reflux control with PPI therapy alone, with <5% requiring subsequent anti-reflux surgery^60^. A significant proportion of patients remain asymptomatic despite abnormal pH monitoring or endoscopic findings, underscoring the need for routine objective surveillance rather than symptom-based assessment alone^62–64^. Given the elevated reflux risk after POEM, surveillance protocols typically include upper endoscopy every 5 years to monitor for oesophagitis and Barrett’s oesophagus. The long-term malignancy risk from chronic GERD after POEM remains uncertain, as the procedure has not been performed in large numbers in the Western world for >10 years. Although post-treatment reflux is common after POEM, data from patients treated with pneumatic dilation and Heller myotomy suggest that Barrett’s oesophagus and oesophageal adenocarcinoma remain rare in achalasia patients despite reflux exposure (annual adenocarcinoma incidence 0.06% across all treatment modalities)^65^.

Beyond POEM-related reflux, the primary malignancy concern in achalasia is squamous cell carcinoma, which arises independently of treatment. Achalasia patients have a substantially elevated baseline risk for oesophageal squamous cell carcinoma, with standardized incidence ratios exceeding 100 and approximately a five-fold increase in overall oesophageal cancer risk compared with the general population^65,66^. This elevated risk, with an estimated annual incidence of 0.18% for squamous cell carcinoma, is attributed to chronic food stasis leading to bacterial overgrowth, fermentation, and persistent mucosal inflammation that can progress to malignancy^8,65^. Risk is particularly elevated in male patients and increases with disease duration, especially after 10–20 years, persisting after treatment with pneumatic dilation, myotomy, or POEM^66–68^. Recent evidence indicates that oesophageal *Candida* infection, present in approximately 12% of achalasia patients, is associated with an eight-fold increased cancer risk and warrants heightened surveillance^68^. Therefore, surveillance endoscopy should include careful inspection for squamous malignancy and oesophageal candidiasis, particularly in male patients with long-standing disease.

### POEM with fundoplication (POEM + F)

To address post-POEM GERD, Inoue *et al*.^69^ introduced POEM + F, a single-session procedure that mimics laparoscopic Heller myotomy with Dor fundoplication. After standard anterior POEM, the peritoneum overlying the anterior gastric wall is dissected and incised to access the peritoneal cavity. Under double-endoscope visualization, the gastric fundus is retracted into the tunnel to simulate a wrap. An endoloop is anchored to the gastric fundus and the distal myotomy edge using endoclips, then progressively tightened to create a partial anterior fundoplication^69,70^. Early results demonstrate promising outcomes. A randomized sham-controlled trial by Maydeo *et al*.^71^ showed that 69% of POEM + F patients achieved oesophageal acid exposure time <6% at 3 months compared with 10% in sham controls (*P* < 0.001), with significant improvements in DeMeester scores and reflux episodes. In a matched cohort study with 3-year follow-up, Bapaye *et al*.^72^ reported objective GERD (acid exposure >6% and/or Los Angeles grade B + oesophagitis) in 13.3% of POEM + F patients *versus* 58.3% of POEM-only patients (*P* = 0.037). Wrap integrity was maintained in approximately 76.5% at 3 years. The safety profile appears favourable, with predominantly minor adverse events, including mucosal injury (8.8%), capnothorax (15%, mostly self-resolving), and clip-related ulceration at the gastro-oesophageal junction (17.6%)^72,73^. Approximately 60% of patients were off PPIs at 1-year follow-up^73^. Although POEM + F shows promise in reducing post-POEM GERD rates, longer-term data and larger multicentre trials are needed to establish the role of POEM + F and determine optimal patient selection. Additionally, reinterventions for refractory GERD after POEM + F have not been reported, and the durability of GERD control beyond 3 years remains unknown. The procedure remains technically demanding and should be performed only by experienced operators^70,74^.

## Comparative effectiveness of primary therapies

RCTs over the past two decades provide high-quality evidence comparing achalasia treatments (*Table 1*)^36,37,42,51,61,75–83^. The randomized trial by Ponds *et al*.^51^ randomized 133 treatment-naïve patients to either POEM or pneumatic dilation. Clinical success at 2 years was achieved in 92% of POEM patients *versus* 54% of those undergoing pneumatic dilation, with POEM demonstrating superiority across all achalasia subtypes, albeit with a higher prevalence of reflux. Werner *et al*.^42^ conducted a multicentre trial comparing POEM with Heller myotomy in 221 patients. Clinical success at 2 years was comparable at 83% for POEM and 82% for Heller myotomy, establishing POEM as non-inferior to the surgical ‘gold standard’. However, reflux oesophagitis was more common with POEM (44% *versus* 29% with Heller myotomy). The European Achalasia Trial provided long-term data comparing pneumatic dilation with Heller myotomy, showing similar clinical success at 5 years (82% *versus* 84%), though multiple dilations were required in the pneumatic dilation group to achieve these outcomes^36^.

**Table 1:** RCTs comparing treatments for achalasia

Study	Year	Comparison	Sample size	Follow-up (years)	Primary outcome	Comparative effectiveness	Adverse events
**POEM *versus* LHM**							
Werner *et al*.^42^	2019	POEM *versus* LHM/Dor	POEM: 112 LHM: 109	2	Eckardt score ≤3 without retreatment	POEM non-inferior to LHM (83% *versus* 82%)	POEM: higher reflux oesophagitis at 2 years (44% *versus* 29%; *P* = 0.023). LHM: mucosal tears 3%
de Moura *et al*.^75^	2022	POEM *versus* LHM/partial fundoplication	POEM: 20 LHM: 20	1	Symptom improvement (change in Eckardt score from baseline)	No significant differences in symptom improvement at 1, 6, and 12 months. Both equally effective	No significant difference in adverse events (3 mucosal tears in POEM group and 1 empyema requiring thoracotomy in LHM group). POEM has significantly higher reflux oesophagitis at all time points (*P* < 0.01)
Hugova *et al*.^61^	2025	POEM *versus* LHM/Dor	POEM: 90 LHM: 87	5	Eckardt score ≤3 without retreatment	Both treatments equally effective (75% *versus* 71%)	POEM: significantly higher reflux oesophagitis (Los Angeles grade B, C, or D) (14% *versus* 7%) and abnormal acid exposure (62% *versus* 31%). More patients on daily PPI with POEM
**LHM *versus* PD**							
Kostic *et al*.^76^	2007	LHM/Toupet *versus* PD	LHM: 25 PD: 26	1	Treatment failure (composite endpoint: inadequate symptom control requiring retreatment or intervention crossover from complications, patient dissatisfaction, clinical decision)	Significant difference in treatment failure (*P* = 0.04), no significant difference in Watson dysphagia scores	PD: two perforations requiring surgery
Boeckxstaens *et al*.^37^	2011	LHM/Dor *versus* PD	LHM: 106 PD: 95	2	Eckardt score ≤3	No significant difference (90% *versus* 86%)	Perforations: PD 4% *versus* LHM 12%. Abnormal acid exposure: PD 15% *versus* LHM 23%
Hamdy *et al*.^77^	2015	LHM *versus* PD	LHM: 23 PD: 25	1	Symptom relief (Demeester dysphagia grading)	LHM more effective clinically and manometrically. Symptom relief: 96% *versus* 76%. LOS pressure significantly lower in LHM group (*P* = 0.0001)	Perforations: PD 8% *versus* LHM 4%. Reflux symptoms: PD 28% *versus* LHM 16% at 1 year
Persson *et al*.^78^	2015	LHM/Toupet *versus* PD	LHM: 25 PD: 28	5	Treatment failure (composite endpoint: inadequate symptom control requiring retreatment or intervention crossover from complications, patient dissatisfaction, clinical decision)	LHM superior to PD (8% *versus* 36% failure; *P* = 0.016). Dysphagia improvement favoured LHM at 3 years but not at 5 years	Not reported
Moonen *et al*.^36^	2016	LHM/Dor *versus* PD	LHM: 105 PD: 96	5	Eckardt score ≤3	Similar success rates (84% *versus* 82%). Redilation performed in 24 (25%) of PD patients	Perforations: PD 5% *versus* LHM 11%. Reflux oesophagitis: PD 14% *versus* LHM 18% at 4 years
Sediqi *et al*.^79^	2021	LHM *versus* PD	LHM: 20 PD: 23	≥10	Treatment failure (composite endpoint: inadequate symptom control requiring retreatment or intervention crossover from complications, patient dissatisfaction, clinical decision)	LHM superior over long-term follow-up (20% *versus* 57% treatment failures)	Not reported
Boeckxstaens *et al*.^80^	2024	LHM/Dor *versus* PD	LHM: 40 PD: 36	10	Eckardt score ≤3	PD and LHM equally effective (74% both groups). Subgroup analysis showed PD was superior to LHM for type II achalasia (*P* = 0.03) while there was a trend that LHM performed better for type III achalasia (*P* = 0.05)	Reflux oesophagitis: LHM 17% *versus* PD 9% at 10 years
**POEM *versus* PD**							
Ponds *et al*.^51^	2019	POEM *versus* PD	POEM: 67 PD: 66	2	Eckardt score ≤3, no retreatment, no severe complications	POEM superior to PD (92% *versus* 54%)	One perforation after PD, no serious adverse events after POEM. Reflux oesophagitis: POEM 41% *versus* PD 7% (*P* = 0.002)
Kuipers *et al*.^81^	2022	POEM *versus* PD	POEM: 62 PD: 63	5	Eckardt score ≤3, no retreatment, no severe complications	POEM superior to PD (81% *versus* 40%). More retreatment needed in PD group (48% retreated)	POEM: higher daily PPI use (46% *versus* 13%; *P* = 0.008) and trend towards more reflux oesophagitis (33% *versus* 13%; *P* = 0.19)
Saleh *et al*.^82^	2023	POEM *versus* PD after failed LHM (rescue therapy)	POEM: 45 PD: 45	1	Eckardt score ≤3 without retreatment	POEM superior for rescue therapy after failed LHM (62% *versus* 267%; *P* = 0.001; OR 0.22). POEM had better emptying on TBO	POEM: one perforation (treated with antibiotics, resolved). PD: one patient required Toupet fundoplication for severe reflux. Reflux oesophagitis: POEM 34% *versus* PD 15% (not significant)
**BT *versus* LHM**							
Zaninotto *et al*.^83^	2004	BT *versus* LHM/Dor or floppy Nissen	BT: 40 LHM: 40	2	Symptom-free	At 6 months both comparable. At 2 years: LHM 88% *versus* 34% symptom-free (*P* < 0.05). Symptoms recurred in 65% of BT-treated patients	LHM: one patient with bleeding from the trocar site, requiring surgery. BT: no complications

POEM, peroral endoscopic myotomy; LHM, laparoscopic Heller myotomy; PPI, proton pump inhibitor; PD, pneumatic dilation; BT, botulinum toxin.

**Fig. 3:**
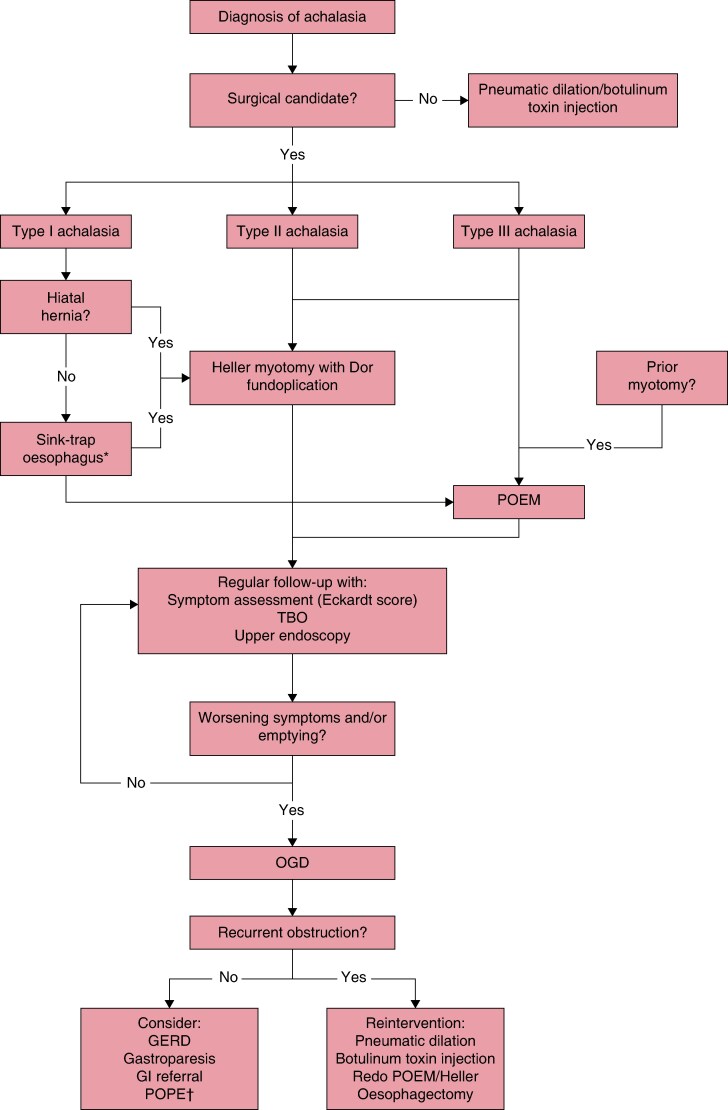
Treatment algorithm for achalasia Algorithm for primary treatment selection and management of recurrent disease. *Sink-trap oesophagus: advanced sigmoid morphology characterized by severe oesophageal angulation and tortuosity resulting in a sharply curved, S-shaped configuration that precludes optimal endoscopic visualization for POEM. †POPE indicated for: massively dilated/tortuous/sigmoid oesophagus with sump formation; failed prior myotomy; and confirmed adequate LOS opening by impedance planimetry. POEM, peroral endoscopic myotomy; TBO, timed barium oesophagram; OGD, oesophagogastroduodenoscopy; GERD, gastro-oesophageal reflux disease; GI, gastrointestinal; POPE, peroral plication of the oesophagus.

Although these trials provide comparative efficacy data in controlled settings, the heterogeneity of achalasia phenotypes and patient characteristics necessitates individualized decision-making. European consensus guidelines from United European Gastroenterology (UEG), the European Society of Neurogastroenterology and Motility (ESNM), and the European Association of Endoscopic Surgery (EAES) strongly recommend that graded repeated pneumatic dilation, Heller myotomy, and POEM demonstrate comparable overall efficacy^32^. These guidelines further recommend individualizing treatment based on patient characteristics, preferences, age, and manometric subtype, as well as institutional expertise^32^. Both POEM and Heller myotomy are effective for type I and type II achalasia^6,25,26^. For type I patients undergoing Heller myotomy, partial fundoplication does not appear to impede gravity-dependent oesophageal emptying in the setting of absent motility^24^. For type III achalasia, POEM is generally favoured over Heller myotomy, and the American Society for Gastrointestinal Endoscopy (ASGE) and SAGES guidelines endorse POEM as the preferred approach for this subtype^7,38^. Patients with a sink-trap oesophagus (that is advanced sigmoid morphology characterized by severe oesophageal angulation and tortuosity resulting in a sharply curved, S-shaped configuration) are generally better served by Heller myotomy, as severe oesophageal angulation precludes optimal endoscopic visualization required for POEM. There is a prevailing belief that elderly or significantly co-morbid patients may benefit from less invasive approaches, including POEM, pneumatic dilation, or botulinum toxin injection. Extensive prior abdominal surgery favours POEM to avoid adhesions. Concurrent hiatal hernia necessitates Heller myotomy to allow for simultaneous repair. Similarly, the presence of an epiphrenic diverticulum generally directs treatment toward Heller myotomy to allow for concurrent diverticulectomy. *Figure 3* summarizes the treatment algorithm for index therapy selection and management of recurrent disease based on these patient and disease factors.

## Management of treatment failure and recurrent disease

Symptoms of achalasia can recur after initial therapy, with up to 20% of patients experiencing recurrent symptoms after myotomy^84^. Myotomy failure may result from incomplete myotomy or intramural scarring. After Heller myotomy, fundoplication-related complications include tight fundoplication, wrap herniation, and narrow crural closure. The timing of recurrence often indicates the underlying cause. Early recurrence of dysphagia (within 12–18 months) typically reflects incomplete myotomy—particularly on the gastric side where the myotomy is technically more difficult—or an excessively tight fundoplication wrap^8^. In the Zaninotto *et al*.^85^ series, failures occurred at a median of 5 (range 1–12) months, with the majority attributed to incomplete myotomy. Late recurrence (beyond 18–24 months) more commonly represents progressive fibrosis, myotomy scarring, or disease progression to sigmoid morphology, and often requires more complex reintervention^86^. Given the risk of recurrence, patients require regular clinical follow-up with symptom assessment. Physiological evaluation with TBO is recommended to complement symptom-based monitoring and objectively monitor treatment durability^8,32,87,88^. When symptoms recur or TBO shows worsening emptying, evaluation should begin with upper endoscopy to exclude obstructive fundoplication (after Heller myotomy) and assess the LOS. The role of post-myotomy manometry remains uncertain; although theoretically useful for evaluating myotomy completeness, evidence suggests routine use has limited utility, as residual high integrated relaxation pressure often fails to correlate with clinical outcomes or symptom recurrence^89,90^. This multimodal evaluation guides selection of appropriate salvage therapy.

Pneumatic dilation offers a less invasive approach for recurrent symptoms after myotomy, avoiding reoperation in distorted tissue planes. Despite theoretical concerns regarding perforation risk, available evidence demonstrates both safety and efficacy^91–96^. A systematic review of 87 patients with failed Heller myotomy undergoing pneumatic dilation (a mean of 2.5 dilations over the span of 26 months) reported 89% success with minimal complications and no perforations^96^. Notably, no perforations after pneumatic dilation were reported in observational studies of patients with prior myotomy^91–95^. Limited data after POEM failure suggest pneumatic dilation remains safe, though efficacy may be modest^97,98^.

POEM has emerged as a safe and effective salvage therapy for failed Heller myotomy, though with outcomes not as good as index POEM. In a multicentre study comparing outcomes of POEM in 90 patients with prior Heller myotomy *versus* 90 treatment-naïve patients, clinical success at a median follow-up of 8.5 months was achieved in 81% of prior surgery patients *versus* 94% of treatment-naïve patients, with no significant differences in adverse events or reflux between groups^99^. A propensity-match study compared 62 patients with prior Heller myotomy undergoing salvage POEM with 62 treatment-naïve patients undergoing index POEM over a median follow-up of 3 years^60^. While operative parameters and complications were comparable between groups, longitudinal symptom palliation differed significantly: at 5 years, 67% of salvage POEM patients *versus* 80% of index POEM patients maintained an Eckardt score of ≤3 (*P* = 0.028). Complete oesophageal emptying at 5 years was achieved in 20% of salvage POEM patients *versus* 24% of index POEM patients (*P* = 0.61). Reinterventions tended to be more frequent after salvage POEM, with constant annual risk after the first year of 5.3% *versus* 2.7% in index POEM patients. The majority of reinterventions after salvage POEM were pneumatic dilations.

The only randomized trial directly comparing salvage interventions by Saleh *et al*.^82^ evaluated POEM *versus* pneumatic dilation in 90 patients with failed Heller myotomy, defined as an Eckardt score of >3 with persistent oesophageal stasis (≥2 cm barium column height at 2 min). Treatment success—defined as an Eckardt score of ≤3 without unscheduled retreatment at 1 year—was significantly higher with POEM than pneumatic dilation (62.2% *versus* 26.7%; *P* = 0.001), though with a trend toward a higher frequency of reflux oesophagitis (34.3% *versus* 15%; *P* = 0.062), which was predominantly mild (grade A–B) in both groups. The lower treatment success with pneumatic dilation compared with earlier observational studies likely reflects the stricter outcome definition (requiring no unscheduled retreatment) and objective inclusion criteria. Current evidence suggests that POEM may be the preferred initial reintervention for failed Heller myotomy. Pneumatic dilation remains a reasonable option for patients unsuitable for or declining POEM, or as salvage therapy after failed salvage POEM. Patients should be counselled that salvage interventions achieve lower success than primary treatment, with the potential need for sequential therapies.

## End-stage achalasia

End-stage achalasia lacks a consensus definition, but is generally characterized by severe oesophageal dilation (width >6 cm) and anatomic distortion; most patients have failed prior interventions, and the concepts of end-stage and refractory achalasia often overlap in clinical practice^6^. Regardless of definition, these patients carry a high risk of aspiration, pneumonia, and malnutrition^100^. Traditional surgical and endoscopic treatments (pneumatic dilation, Heller myotomy, or POEM) are often ineffective in megaoesophagus or sigmoid oesophagus and carry increased risk. For example, POEM in sigmoid oesophagus is associated with a two-fold increase in periprocedural complications^99^. Although conservative approaches such as repeat myotomy may benefit selected patients^98,101,102^, many with end-stage disease ultimately require definitive intervention with oesophagectomy.

The peroral plication of the oesophagus (POPE) procedure is an emerging endoscopic alternative to oesophagectomy for select patients with end-stage achalasia. The procedure utilizes endoscopic suturing to create full-thickness plications that narrow the dilated oesophagus and eliminate distal oesophageal ‘sump’ formation—areas where food and secretions pool due to oesophageal redundancy and sigmoid morphology^103^. POPE is performed using a dual-chamber endoscope fitted with an endoscopic suturing device to place full-thickness plication sutures in a distal-to-proximal manner along redundant oesophageal segments^103,104^. The plications straighten the oesophagus and collapse the sump, retubularizing the organ to restore a more anatomically favourable configuration that allows gravity to aid oesophageal emptying^103,104^. Patient selection for POPE requires careful evaluation to identify those whose symptoms are primarily due to anatomic oesophageal redundancy and sump formation rather than outlet obstruction alone^103,104^. Key inclusion criteria in case series include: massively dilated, tortuous, or sigmoid oesophagus with distal oesophageal sump formation visible on barium oesophagram; prior failed interventions (pneumatic dilation, Heller myotomy, POEM); and confirmation by impedance planimetry that prior myotomies achieved adequate LOS opening^103,104^. Patients with untreated achalasia should undergo myotomy first before POPE is considered^103^. The procedure is contraindicated when symptoms are primarily due to LOS dysfunction or when imaging does not demonstrate clear anatomic abnormality (redundancy, sump formation) to explain symptoms^103^.

Early case series demonstrate promising short-term results, with 82–90% of achalasia patients experiencing symptom improvement at initial follow-up^103,104^. Objective improvement has been demonstrated on TBO, with reduction in oesophageal column height and elimination of sump formation^104,105^. The safety profile is favourable, with no deaths or serious intraoperative complications reported, and most procedures require 60–90 min with same-day or next-day discharge^103,104^. However, POPE is most effective for patients whose symptoms are primarily due to anatomic oesophageal redundancy and sump formation rather than outlet obstruction alone, and approximately 30–50% of patients require repeat procedures in long-term follow-up^103,104^. The durability of plication remains under investigation. Although the procedure’s repeatability and favourable morbidity profile present advantages for delaying oesophagectomy in high-risk patients, the procedure’s role as a definitive long-term option has not been established. Furthermore, the altered luminal anatomy after oesophageal plication raises concerns regarding oncological surveillance; given the elevated baseline risk of squamous cell carcinoma in end-stage achalasia, the feasibility and adequacy of endoscopic surveillance in a plicated oesophagus remains an important unanswered question that warrants consideration when selecting candidates for this procedure.

Despite this emerging endoscopic option, oesophagectomy often provides the greatest symptom relief for end-stage achalasia, despite the significant risks and impact on quality of life^106,107^. However, oesophagectomy should not be performed on the basis of anatomy alone as every patient should be considered for myotomy. Patient selection is critical; the risks and life-altering consequences of oesophagectomy must be clearly outweighed by the expected benefit. Candidates must be profoundly symptomatic, with recurrent aspiration pneumonia, failure to thrive, or significantly impaired quality of life constituting the primary indications, the last being most common. Observational studies of oesophagectomy for end-stage achalasia report relatively high incidences of postoperative respiratory complications, but relatively low mortality in carefully selected patients at high-volume centres^107^. At 12–75-month follow-up, 75–100% of patients resume an unrestricted diet^107^, with one study reporting a median weight gain of 6.3 kg at 72 months^108^.

Several operative considerations distinguish oesophagectomy for achalasia from oncological resection, though, given the rarity of this indication, high-quality comparative data to guide operative technique are limited, and the following recommendations reflect expert opinion and institutional experience. Anaesthetic induction requires particular caution given the risk of aspiration from retained food and secretions in the dilated oesophagus. Because this is a benign indication, the absence of neoadjuvant chemotherapy or radiotherapy works in the surgeon’s favour, as tissue planes are preserved and healing is not compromised. Similarly, extensive lymphadenectomy is unnecessary and should be avoided to minimize the risk of chylothorax. While transhiatal oesophagectomy was historically performed in this setting, minimally invasive transthoracic approaches have become increasingly preferred and have largely replaced it. Thoracoscopic mobilization is preferred over open transthoracic dissection, as mobilizing a massively dilated oesophagus increases the risk of airway injury; furthermore, the dilated oesophagus can distort mediastinal anatomy and displace the recurrent laryngeal nerve from the usual position, making direct visualization essential. Maximizing thoracoscopic dissection in the chest is therefore recommended to reduce the risk of right recurrent laryngeal nerve injury during the cervical portion of the operation. Gastric conduit creation using a stomach with prior fundoplication may limit conduit reach. Surgeons should anticipate the dilated proximal oesophagus, which creates size mismatch challenges for anastomotic techniques. A cervical anastomosis, as performed in a McKeown three-stage approach, is generally preferred over an intrathoracic anastomosis (that is Ivor-Lewis) because the cervical oesophagus tends to be less dilated than the intrathoracic oesophagus. A hand-sewn anastomosis is preferred over a stapled anastomosis to accommodate luminal size discrepancy. Conduit redundancy should be avoided to prevent delayed emptying and symptom recurrence. In summary, oesophagectomy is recommended for patients with megaoesophagus who have failed other interventions and whose symptoms substantially impair quality of life, and should be performed at high-volume centres experienced in the unique technical demands of benign end-stage disease.

## Role of the endoluminal functional lumen imaging probe (EndoFLIP) in achalasia management

EndoFLIP is a catheter-based device that uses impedance planimetry to measure real-time cross-sectional area and distensibility of the oesophagogastric junction during controlled balloon distention. Unlike high-resolution manometry, which measures pressure, EndoFLIP directly assesses the physical opening and compliance of the oesophagogastric junction—measurements that more directly reflect oesophageal emptying. The role of EndoFLIP in achalasia management is anticipated to expand, with applications in diagnosing achalasia, guiding therapy, and monitoring treatment response. The American Gastroenterological Association identifies intraoperative guidance as a key area for growth, with real-time EndoFLIP measurements during myotomy (POEM or minimally invasive Heller myotomy) to guide adequacy of LOS disruption^26,109^. Data from meta-analyses demonstrate that EndoFLIP-guided myotomy correlates with improved oesophagogastric junction distensibility, greater symptomatic response, and reduced rates of reflux oesophagitis compared with procedures performed without intraoperative guidance^110^. Beyond intraoperative applications, EndoFLIP demonstrates utility in post-treatment surveillance, as changes in oesophagogastric junction distensibility and diameter correlate with clinical response and oesophageal emptying on TBO^111^. Additionally, the ability of EndoFLIP to detect contractile patterns not visualized by high-resolution manometry may enable subclassification of achalasia and personalized treatment strategies, while serving as a practical diagnostic alternative for patients unable to tolerate manometry.

However, optimization of EndoFLIP utilization requires ongoing research to standardize protocols, define optimal distensibility index thresholds, and establish procedural endpoints that maximize clinical outcomes while minimizing adverse effects. As evidence accumulates regarding the predictive value of EndoFLIP for treatment response and the complementary role of EndoFLIP to high-resolution manometry in diagnostically ambiguous cases, this technology is poised to become an integral component of comprehensive achalasia management—from initial diagnosis through intraoperative decision-making to long-term follow-up.

## Conclusion

Optimal achalasia treatment requires individualization based on patient characteristics, disease subtype, oesophageal morphology, and institutional expertise. Pneumatic dilation remains a valuable option in patients who are suboptimal candidates for a myotomy, but long-term outcomes are inferior to Heller myotomy or POEM for primary therapy. POEM and minimally invasive Heller myotomy, typically with fundoplication, represent first-line therapies for most patients, offering similar improvements in symptoms and oesophageal emptying. While POEM demonstrates higher rates of GERD, most patients achieve control with PPIs; however, long-term Barret’s oesophagus and cancer risk from long-term post-POEM reflux (>10–15 years) remain undetermined. Treatment selection should be guided by institutional expertise and available resources. Although many technical skills required for Heller myotomy can be adapted from general foregut surgery experience, POEM requires distinct technical proficiency without direct analogous procedures for skill transfer. For recurrent disease, POEM provides effective salvage therapy after failed Heller myotomy, though outcomes tend to be inferior to those of index POEM. In highly selected patients with end-stage disease, oesophagectomy can be performed with low morbidity at high-volume centres. Despite achalasia being a chronic, progressive disorder, contemporary endoscopic and surgical treatment can achieve excellent long-term quality of life. As the field continues evolving with emerging technologies and longer follow-up data, treatment algorithms will be further refined to optimize patient outcomes.

## Supplementary Material

znag046_Supplementary_Data

## Data Availability

All data used in this review are derived from previously published studies, and the original data are publicly available through the respective cited sources.
