# Supplementary material for: Modern treatment for achalasia: endoscopic and surgical therapies
Source: Br J Surg. 2026 Apr 27;113(5):znag046. doi: 10.1093/bjs/znag046 (PMC13177705; doi:10.1093/bjs/znag046)
Supplement: znag046_Supplementary_Data [file znag046_supplementary_data.docx]

| **Supplementary Table 1. The Eckardt Score for Achalasia Symptom Severity** | | | | |
| --- | --- | --- | --- | --- |
| **Score** | **Dysphagia** | **Regurgitation** | **Chest Pain** | **Weight Loss (kg)** |
| 0 | None | None | None | None |
| 1 | Occasional | Occasional | Occasional | <5 |
| 2 | Daily | Daily | Daily | 5-10 |
| 3 | Each meal | Each meal | Each meal | >10 |
| Total score range: 0-12. Each domain scored 0-3. Treatment success/remission is typically defined as total score ≤3. | | | | |
